# Effectiveness of Virtual Reality-Based Training Versus Conventional Exercise Programs on Fall-Related Functional Outcomes in Older Adults with Various Health Conditions: A Systematic Review

**DOI:** 10.3390/jcm14155550

**Published:** 2025-08-06

**Authors:** Krzysztof Kasicki, Ewa Klimek Piskorz, Łukasz Rydzik, Tadeusz Ambroży, Piotr Ceranowicz, Maria Belcarz Ciuraj, Paweł Król, Wiesław Błach

**Affiliations:** 1 Department of Physiotherapy, Faculty of Health Sciences, Collegium Medicum, Andrzej Frycz-Modrzejewski Krakow University, 30-705 Kraków, Poland; 2 Department of Rehabilitation in Rheumatology and Geriatrics, Institute of Clinical Rehabilitation, Faculty of Physical Rehabilitation, University of Physical Culture, 31-571 Kraków, Poland; ewa.klimek@awf.krakow.pl; 3 Institute of Sports Sciences, University of Physical Culture, 31-571 Kraków, Poland; lukasz.rydzik@awf.krakow.pl (Ł.R.); tadek@ambrozy.pl (T.A.); 4 Department of Physiology, Faculty of Medicine, Jagiellonian University Medical College, 31-531 Kraków, Poland; piotr.ceranowicz@uj.edu.pl; 5 Department of Nursing, Faculty of Health Sciences, Vincent Pol University, 20-816 Lublin, Poland; maria.belcarz@gmail.com; 6 Institute of Physical Culture Sciences, College of Medical Sciences, University of Rzeszów, 35-959 Rzeszów, Poland; pkrol@ur.edu.pl; 7 Faculty of Sport, University School of Physical Education in Wroclaw, 51-612 Wrocław, Poland; wieslaw.judo@wp.pl

**Keywords:** fall risk, training, elderly, VR, multicomponent training

## Abstract

**Background/Objectives:** The aim of this systematic review was to compare the effectiveness of virtual reality (VR)-based training with conventional exercise programs in improving functional outcomes related to fall risk among older adults with various health conditions. **Methods:** The review was conducted in accordance with the PRISMA 2020 guidelines and registered in PROSPERO (registration number CRD42022345678). The databases Scopus, PubMed, Web of Science, and EBSCO were searched up to 31 March 2025. Randomized controlled trials (RCTs) were included if they involved participants aged ≥60 years, a VR intervention lasting ≥6 weeks, and a control group performing traditional exercises or receiving usual care. Methodological quality was assessed using the PEDro scale, and a narrative synthesis was performed across four outcome domains: balance, mobility, cognitive function, and fall risk. **Results:** Seven RCTs were included in the analysis (totaling 664 participants). VR training was found to be at least as effective as conventional exercise in improving balance (e.g., Berg Balance Scale) and mobility (e.g., Timed Up and Go), with some studies showing superior effects of VR. One RCT demonstrated that combining VR with balance exercises (MIX) yielded the greatest improvements in muscle strength and physical performance. Additionally, two studies reported cognitive benefits (e.g., MoCA) and a 42% reduction in fall incidence within six months following VR intervention. The methodological quality of the included studies was moderate to high (PEDro score 5–9/10). **Conclusions:** VR-based training represents a safe and engaging supplement to geriatric rehabilitation, effectively improving balance, mobility, and, in selected cases, cognitive function, while also reducing fall risk.

## 1. Introduction

The ageing population is undoubtedly one of the most significant challenges facing modern healthcare and the broader economic outlook. According to the literature, by the year 2050, the number of individuals over the age of 65 is projected to constitute approximately 16% of the global population [1]. The global prevalence of falls among older adults is estimated at around 26.5% [2]. In 2017 alone, approximately 8.4 million medical consultations were reported in Western Europe due to fall-related injuries in adults aged 70 or older [3]. This issue is serious not only due to the growing number of incidents but also because of the increasing financial burden on healthcare systems caused by the need for prolonged hospitalisation. For example, in the United States, data collected for 2015 indicated an annual expenditure of about USD 50 billion by the healthcare system, of which 28.9 billion was attributed to non-fatal fall-related costs [4,5]. It is an undeniable fact that these correlations are closely linked to population ageing and the growing proportion of individuals aged over 60 in the general demographic.

Age-related diseases encompass metabolic, cardiovascular, neurodegenerative, and musculoskeletal conditions. Undoubtedly, ageing is the most critical risk factor for developing neurodegenerative disorders, with Alzheimer’s disease being the most prevalent of its kind worldwide [6]. Parkinson’s disease follows, characterised by the loss of dopaminergic neurons in the substantia nigra [7]. The incidence of Parkinson’s disease increases up to tenfold between the ages of 50 and 80 [8]. Notably, the progressive decline in brain function due to ageing is associated with reduced learning and memory capabilities [9]. Ageing also affects cardiovascular tissues, leading to increased arterial stiffness, endothelial dysfunction, and myocardial hypertrophy. In older individuals, cardiomyocytes—responsible for contraction and relaxation—decrease in number but increase in size [10]. These changes significantly contribute to the development of stroke, hypertension, atherosclerosis, and myocardial infarction [11]. Furthermore, ageing is associated with a reduction in maximum heart rate and cardiac output, leading to impaired cardiac reserve and, consequently, heart failure, fatigue, and reduced exercise tolerance [12]. In the immune system, age-related changes can weaken or impair the body’s ability to combat infections and cancer, increasing the risk of autoimmune disorders [13,14].

The musculoskeletal system, which plays a key role in safe mobility, balance, and fall prevention, is primarily affected by osteoarthritis and sarcopenia. In a study by Xu et al., the authors compared comorbidities with fall risk [1]. They found that cognitive impairments and diabetes were not significantly associated with fall risk, in contrast to Parkinson’s disease, cardiovascular conditions, hypertension, and general frailty, which were linked to an increased likelihood of falling in individuals over 60 years of age [1].

The literature reports that older adults with poor balance, mobility, or neurosensory risk factors are 33–51% more likely to experience recurrent falls [15]. Functional capacity results from the integration of multiple motor abilities, and its decline directly translates into increased fall risk. Balance—both static and dynamic—plays a critical role and results from the interplay between the nervous, muscular, and sensory systems, including vision, proprioception, and the vestibular apparatus [16,17,18,19]. Ageing leads to a progressive decline in these systems, manifesting as reduced postural stability, delayed response to sudden positional changes, and impaired gait control. Muscle strength, especially in the lower limbs and postural muscles, is another key factor; its reduction impairs basic daily functioning [20]. Motor coordination—referring to the precise and synchronised activity of various muscle groups—is essential for avoiding hazards such as tripping or slipping. Additionally, reduced endurance and diminished cardiovascular fitness lead to faster fatigue, further increasing fall risk during prolonged or demanding physical activities [21,22]. Cognitive function, particularly executive function and attention, also plays a crucial role in responding to unexpected situations [23]. Therefore, the complexity of fall risk in older adults necessitates a comprehensive approach, integrating motor, cognitive, and sensory domains in the development of effective fall prevention programs.

Exercise programs play a key role in fall prevention by improving strength and balance, thereby reducing fall risk by approximately 20–30% [24]. Unfortunately, traditional exercise programs often suffer from low attractiveness and poor adherence among many older adults [24]. Virtual reality (VR)-based training has emerged as an alternative approach, combining rehabilitation with elements of gamification. Previous meta-analyses have confirmed, among other findings, that VR-based interventions more effectively improve balance and reduce fear of falling compared to no intervention [25]. Some analyses have even suggested the superiority of VR over conventional exercises—for example, showing greater improvements in functional mobility among healthy older adults or greater increases in balance test scores in VR groups compared to standard exercise groups [26,27]. However, there is still no consensus on whether VR significantly outperforms conventional training across all older populations and outcome domains. Existing reviews have certain limitations—many focused on selected groups (e.g., only healthy adults aged 60+, or only individuals with Parkinson’s disease) or compared VR to passive controls [25,27,28]. In recent years, growing attention has been paid to innovative training modalities based on virtual reality (VR), which may serve as engaging supplements or alternatives to traditional methods [29]. VR training involves performing motor tasks in an interactive, computer-generated environment that enables safe simulation of daily activities and fall-related situations under controlled conditions [30]. This type of training frequently incorporates simultaneous visual, auditory, and proprioceptive stimuli, along with the demand for rapid postural responses, supporting the development of balance reflexes and multisensory integration [30]. The effectiveness of VR training is underpinned by similar adaptive mechanisms as in conventional rehabilitation, particularly neuroplasticity [31]. Repeated motor task performance in immersive and variable conditions stimulates neuroplastic changes in the central nervous system, contributing to lasting improvements in postural control and reactive balance [32]. As such, VR appears to be a promising tool in fall prevention, thanks to its ability to simultaneously enhance balance, proprioception, and postural responses while increasing patient engagement—ultimately promoting autonomy and functional safety in the aging population.

This review provides an updated and comprehensive synthesis of the evidence, including the most recent randomized controlled trials (RCTs) and a broad population of individuals aged 60 and over—both healthy and those with conditions such as Parkinson’s disease, mild cognitive impairment (MCI), and osteoporosis. It directly compares VR training to conventional exercise programs, thereby allowing for an evaluation of the additional value of VR as a preventive intervention relative to standard methods. The review focuses on key indicators of fall risk: static and dynamic balance, mobility (gait, TUG), cognitive functions (relevant, for example, for dual-tasking), as well as fall frequency and fear of falling. A central feature of this review is its focus on head-to-head comparisons between VR interventions and standard exercise programs. Many earlier studies compared VR primarily to no intervention or placebo (e.g., Neri et al. demonstrated advantages of VR over no intervention, as did Saragih et al.), or they contrasted VR with broadly defined “usual care” [25,33]. This review includes RCTs in which the control group performed conventional physical exercises matched in intensity and duration to the VR group. This allowed the assessment of whether VR provides added value beyond what is achieved with typical physical training. Direct comparisons between these two intervention types have been rare—one of the few examples is a 2022 meta-analysis on healthy older adults, which strictly compared VR to “traditional physical activity” and found VR to be superior in improving mobility and balance [27]. However, that study only included healthy older adults, whereas the present review extends this approach to the general 60+ population, including individuals with various health conditions.

Previous reviews focused primarily on physical parameters (e.g., static and dynamic balance, gait speed, mobility tests) and, in some cases, fear of falling. Therefore, the current review additionally includes cognitive outcomes and fall frequency, provided these were reported in the RCTs. This inclusion was motivated by the observation that VR training frequently engages cognitive processes (e.g., attention, reaction time, dual-tasking), which may contribute to fall prevention—an aspect that has not been comprehensively addressed in prior reviews (an exception being Shen’s 2021 review on MCI, which assessed cognitive function) [34]. An attempt was made to qualitatively discuss the impact of VR on fall frequency over a longer term, aiming to provide a more holistic view of the available findings. Accordingly, the objective of this systematic review is to synthesize evidence regarding the effectiveness of virtual reality (VR)-based training compared to conventional exercise programs in improving functional outcomes related to fall risk in older adults with diverse health conditions.

The following research questions were formulated:

- In which subgroups of older adults (considering different medical conditions) is VR training more effective in reducing fall risk than conventional exercise programs?
- What is the impact of VR interventions and conventional programs on specific motor parameters such as static and dynamic balance, lower limb strength, functional mobility (e.g., Timed Up and Go), and gait characteristics?
- What are the adherence rates, safety indicators, and long-term effects (follow-up) of both types of interventions?
- What practical and clinical factors (e.g., technology acceptance, equipment requirements, therapist competence) influence the implementation of VR in fall prevention programs in geriatric care settings?

## 2. Methods

### 2.1. Search Strategy

The systematic review was conducted in accordance with the Preferred Reporting Items for Systematic Reviews and Meta-Analyses (PRISMA) guidelines. The review protocol was registered in the PROSPERO database under the registration number CRD420251052648. Until 31 March 2025, two of the authors (K.K. and Ł.R.) independently searched four electronic databases—Scopus, Web of Science, PubMed, and EBSCO—to identify relevant studies concerning fall risk and virtual reality (VR) interventions. The following keyword combinations were applied for each database:

- Scopus:
  TITLE-ABS-KEY ((“virtual reality” OR VR OR exergam*) AND (“circuit training” OR circuit-based OR multicomponent OR multimodal OR “combined exercise”) AND (“balance training” OR “isolated balance” OR “postural control” OR “postural stability”) AND (falls OR “fall prevention” OR “fall risk” OR “accidental falls”) AND (“older adults” OR elderly OR aged OR geriatric OR senior OR “community-dwelling”))
- PubMed:
  (“Virtual Reality” [Mesh] OR “virtual reality” [tiab] OR VR [tiab] OR exergam* [tiab]) AND (“Exercise Therapy” [Mesh] OR multicomponent [tiab] OR “combined exercise” [tiab] OR “circuit training” [tiab]) AND (“Accidental Falls” [Mesh] OR fall* [tiab] OR “fall risk” [tiab] OR “fall prevention” [tiab]) AND (“Aged” [Mesh] OR elderly [tiab] OR “older adults” [tiab]) AND (randomized controlled trial [pt] OR clinical trial [pt])
- EBSCO:
  ((“virtual reality” OR VR OR exergam*) AND (“circuit training” OR circuit-based OR multicomponent OR multimodal OR “combined exercise”) AND (“balance training” OR “isolated balance” OR “postural control” OR “postural stability”) AND (falls OR “fall prevention” OR “fall risk” OR “accidental falls”) AND (“older adults” OR elderly OR aged OR geriatric OR senior OR “community-dwelling”))
- Web Of Science:
  TS = (“virtual reality” OR VR OR exergam*) AND TS = (multicomponent OR “combined exercise” OR “circuit training”) AND TS = (fall* OR “fall risk” OR “fall prevention”) AND TS = (elderly OR “older adults”)

### 2.2. PICOS Inclusion and Exclusion Criteria

The inclusion and exclusion criteria were defined according to the PICOS model. Only randomized controlled trials (RCTs) were considered eligible if they included more than 10 participants per group and had an intervention duration of more than 6 weeks. Studies without a control group were excluded. The focus was placed on quantitative parameters related to functional performance or fall risk. Eligible studies were required to implement a virtual reality (VR)-based intervention in any form, while the comparator was defined as either any form of exercise program or no-exercise control. Detailed criteria are presented in Table 1 below.

### 2.3. Selection Process

The selection of eligible studies was conducted by two authors (P.K. and T.A.). First, all records retrieved from the databases were screened, and duplicates as well as articles lacking full-text availability in English were manually removed. Titles and abstracts were re-assessed. Eligibility criteria, based on the PICOS model and outlined in the previous table, were applied. Any discrepancies were discussed until a consensus was reached among all authors. The same strategy was applied during the full-text analysis of the remaining articles. Additionally, the reference lists of the included studies were screened to identify any relevant publications that may have been missed during the initial search. No further articles were identified for inclusion.

### 2.4. Data Extraction

The screening and data extraction process was conducted by two authors (K.K. and E.K.-P.) and included variables such as sample size, study outcomes, intervention duration, type of intervention, and measurement tools. Any discrepancies were discussed with a third author (P.C.), and final decisions were made through consensus. The methodological quality of the included studies was assessed using the Physiotherapy Evidence Database (PEDro) scale (Table 2).

### 2.5. Data Items

The following variables were extracted from each RCT:

- Publication characteristics: authors, year, title, and publication source.
- Study design: type (randomized, controlled/pre–post/cross-sectional), setting, number of participants, and inclusion criteria.
- Participants: age, sex, health status (e.g., idiopathic falls, Parkinson’s disease, mild cognitive impairment [MCI], osteoporosis, chronic dizziness, healthy older adults), and physical activity level.
- VR intervention: type, session duration (30–60 min), frequency (2–5 times/week), and intervention period (3–12 weeks).
- Control intervention: conventional balance exercises (traditional treadmill training, stretching/relaxation), home-based programs (educational materials, balance exercises), or no intervention.
- Balance assessments: Berg Balance Scale, tandem stance, one-leg stance test, Functional Reach Test, Fullerton Advanced Balance Scale.
- Mobility assessments: Timed Up and Go (TUG), 10-Meter Walk Test (10MWT), gait speed, and gait variability.
- Lower limb muscle strength: isokinetic strength of the quadriceps and hamstrings.
- Cognitive function: Montreal Cognitive Assessment (MoCA).
- Fall risk and fear of falling: Falls Efficacy Scale (FES), Physiological Profile Assessment (PPA).
- Dizziness: Vertigo Symptom Scale (VSS) and Dizziness Handicap Inventory (DHI).
- Parkinson’s disease symptoms: Movement Disorders Society–Unified Parkinson’s Disease Rating Scale (MDS-UPDRS), motor section.
- Number of falls: incidence of fall events before and after the intervention (falls per 6 months).

### 2.6. Effect Measures

For each outcome, statistical measures were selected according to the type of data. In the case of quantitative outcomes (e.g., balance scales, TUG time, gait speed, muscle strength), mean differences between groups with confidence intervals or analysis of covariance (ANCOVA) controlling for baseline values were commonly used. For studies reporting falls, the primary measure was fall incidence, expressed as a rate per unit of time, and between-group effects were assessed using the incident rate ratio (IRR) with a 95% confidence interval. In analyses involving VR interventions for Parkinson’s disease or vestibular disorders, standardized clinical scales (e.g., the UPDRS or vestibular disorder scales) were used, and outcomes were reported either as the mean point difference between groups or as a percentage change in scale scores. For binary variables (e.g., fall occurrence: yes/no), odds ratios or relative risk were applied, although most cited studies predominantly reported continuous or count data.

### 2.7. Synthesis Methods

Due to the high heterogeneity of the study populations (various medical conditions) and the interventions applied, the synthesis of results was conducted narratively. The findings from individual studies were thematically grouped (balance, mobility, cognitive function, fall risk) without performing a formal meta-analysis. Consistencies and discrepancies in observed effects were highlighted across studies with similar characteristics. Methodological heterogeneity was described narratively, including analyses of differences in intervention duration and the use of various outcome measures. In cases of missing data (e.g., when certain outcomes were not reported in publications), a qualitative analysis was used as a compensatory strategy: when a single study did not provide a given outcome value, general trends from other sources were considered. Comparisons between intervention groups were performed within similar patient subgroups—studies involving patients with Parkinson’s disease, osteoporosis, or mild cognitive impairment were analysed separately.

### 2.8. Study Risk of Bias Assessment

The PEDro scale and the RoB 2 tool were used to assess the quality of the studies included in the review. Two authors (K.K. and E.K.-P.) independently evaluated the methodological quality of each publication. In cases where the data were insufficiently clear to allow for definitive scoring, a third author (MBC) was consulted to reach a consensus and finalize the decision. The PEDro scale consists of 11 criteria: eligibility, random allocation, concealed allocation, baseline comparability, blinding of participants, therapists, and assessors, dropout rate below 15%, intention-to-treat analysis (ITT), between-group comparisons, and reporting of point estimates with variability measures. Each criterion (except the first, which pertains to external validity and is not scored) is assessed in a binary manner—receiving a score of either 1 or 0. Studies scoring 4–5 points are considered to have moderate methodological quality, while scores above 6 points are interpreted as indicating high-quality studies (Table 2). The RoB 2 tool [35], a revised version of the Cochrane risk-of-bias assessment method, was also used. This tool covers five key domains, allowing for a detailed analysis of potential sources of bias within randomized controlled trials (RCTs).

## 3. Results

### 3.1. Article Identification

**Figure 1:**
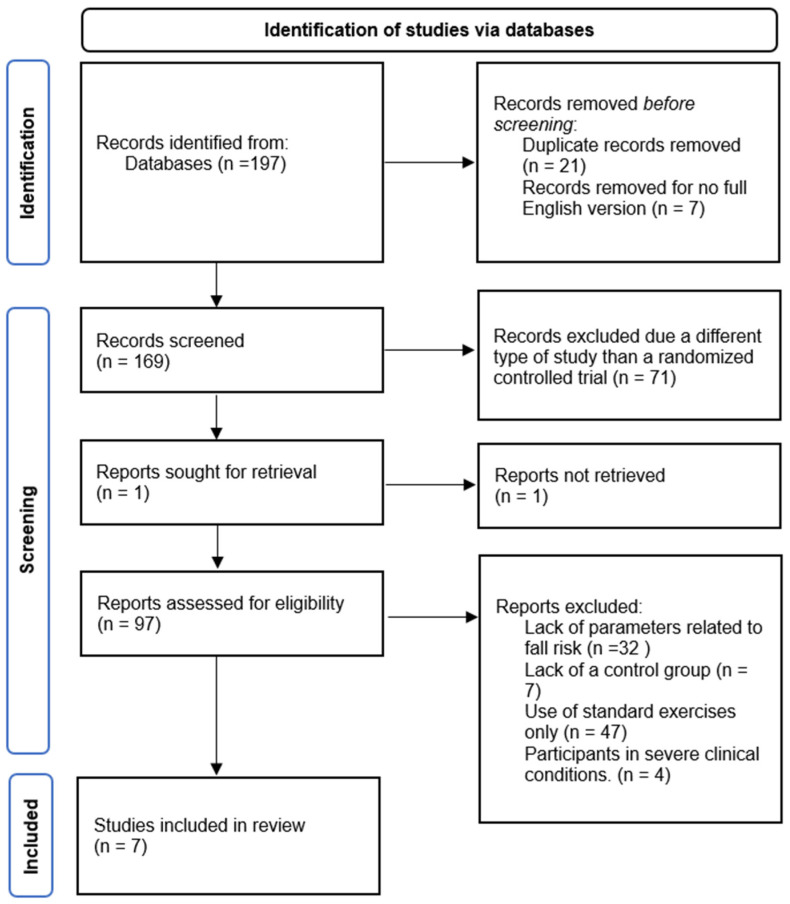
PRISMA flowchart.

Initially, a total of 197 records were identified across the four databases. After removing duplicates, 176 records were manually screened based on abstracts, titles, and keywords. Additionally, seven records were excluded during this phase due to the lack of full-text availability in English. In the next step, studies were assessed for their design, and 71 records that did not meet the criteria of randomized controlled trials were excluded. Ultimately, 97 records were subjected to full-text eligibility assessment according to the predefined PICOS framework. As a result, seven studies met the inclusion criteria and were retained for further analysis. The detailed search and selection process is illustrated in Figure 1.

### 3.2. Study Characteristics

A detailed description of the included studies and a summary of the main findings are presented in Table 3. All seven studies were published between 2016 and 2024 and involved older adult populations with a mean age ranging from 59.3 to 74.2 years. The study by Mirelman et al. (2016) included 302 participants (INT: 154; control: 148), Nicolien M. van der Kolk et al.—130 (INT: 65; control: 65), Kochaphan Phirom et al.—40 (INT: 20; control: 20), Sadeghi et al. (2021)—64 (BT: 16, VR: 16, MIX: 16, CON: 16), Babadi and Daneshmandi (2021)—36 (12 per group), Kanyilmaz et al. (2022)—32 (INT: 16; control: 16), and Yilmaz & Kösehasanoğulları (2024)—60 (INT: 30; control: 30) [36,37,38,39,40,41,42]. The mean age of participants in the respective studies was as follows: 74.2 ± 6.9 years [39], 59.3 ± 8.3 years [36], 70.21 ± 4.18 years [37], 71.8 ± 6.09 years [38], 66.5–67.5 ± 3.1–3.8 years [40], 69.7 ± 6.3 years [41], and 67 ± 10.64 to 68 ± 9.06 years [42]. In studies [36,37,39,40,41], both male and female participants were included; one study enrolled only men [38], and another included only women [42].

The interventions included the following: treadmill training with VR (obstacle and pathway simulation) three times per week for six weeks [39]; aerobic cycling training with VR (50–80% HRR) three times per week for six months [36]; combined physical and cognitive training using Xbox Kinect three times per week for twelve weeks [37]; balance training (BT) and VR either combined (MIX) or applied separately (VR, BT), three times per week for eight weeks [38]; training with Xbox Kinect (sports games) versus conventional balance training, three times per week for nine weeks [40]; VR-supported vestibular rehabilitation using 360° videos in VR goggles, five times per week for three weeks [41]; and balance exercises with Nintendo Wii, three times per week for twelve weeks [42].

Control groups performed traditional training, including treadmill walking without VR [39], stretching/relaxation exercises [36], educational materials and phone follow-ups [37], or received no intervention [38,40,41,42].

The main outcome measures included fall incidence, gait speed and variability, endurance, and cognitive function [39]; severity of Parkinson’s symptoms (MDS-UPDRS III) and VO_2_max [36]; fall risk (PPA, TUG) and cognitive function (MoCA) [37]; muscle strength, mobility, and balance (TUG, 10MWT) [38]; balance (SLS, FRT, FAB, TUG) [40]; dizziness symptoms (VSS, DHI), anxiety and depression (HAS, GDS), and fear of falling (FES I) [41]; and balance (BBS, TUG) and fear of falling (FES) [42].

### 3.3. Risk of Bias in Included Studies

The overall PEDro scores are presented in Table 2. The methodological quality of the seven included studies was as follows: One publication achieved a “high methodological quality” rating with a score of 9 out of 10 points [36]. Two additional studies scored 7/10 and 6/10, respectively, which are also classified as “high methodological quality” [38,39]. The remaining four publications received a score of 5/10, indicating a moderate level of methodological quality [37,40,41,42].

**Table 2:** PEDro scale for the included studies.

Lp.	Study	1. Randomisation	2. Allocation Concealment	3. Baseline Comparability	4. Patient Blinding	5. Therapist Blinding	6. Assessor Blinding	7. ≥85% Follow-Up	8. Intention-to-Treat	9. Between-Group Comparisons	10. Point Measures & Variability	Total Score:
1	Mirelman et al., 2016 [39]	−	+	+	−	−	−	+	+	+	+	7/10
2	van der Kolk et al., 2019 [36]	+	+	+	+	−	+	+	+	+	+	9/10
3	Phirom et al., 2020 [37]	+	−	+	−	−	−	+	−	+	+	5/10
4	Sadeghi et al., 2021 [38]	+	−	+	−	−	+	+	−	+	+	6/10
5	Yousefi Babadi et al., 2021 [40]	+	−	+	−	−	−	+	−	+	+	5/10
6	Kanyılmaz et al., 2022 [41]	+	−	+	−	−	−	+	−	+	+	5/10
7	Yilmaz & Kösehasanoğulları 2024 [42]	+	−	+	−	−	−	+	−	+	+	5/10

Marks: (+), item fulfilled; (−), item not fulfilled.

According to the assessment conducted using the RoB 2 tool, most of the analyzed studies were characterized by low or moderate risk of bias. The following findings were observed across the individual RoB 2 domains:

- Domain 1 (bias arising from the randomization process): Three out of seven studies [40,41,42] were rated as raising some concerns regarding the randomization process. One study [37] was rated as having a high risk of bias in this domain. The remaining two studies [36,39] demonstrated a low risk of bias.
- Domain 2 (bias due to deviations from intended interventions): All studies [36,37,38,39,40,41,42] were rated as having a low risk of bias.
- Domain 3 (bias due to missing outcome data): [36,37,38,39,40,41,42] 100% of the studies showed a low risk of bias.
- Domain 4 (bias in measurement of the outcome): Six studies were rated as having a low risk of bias [36,37,38,39,41,42], while one study [40] was classified as having some concerns.
- Domain 5 (bias in selection of the reported result): Four studies [36,38,39,40] were rated as having a low risk of bias, while the remaining three [37,41,42] were classified as raising some concerns in this domain.

**Figure 2:**
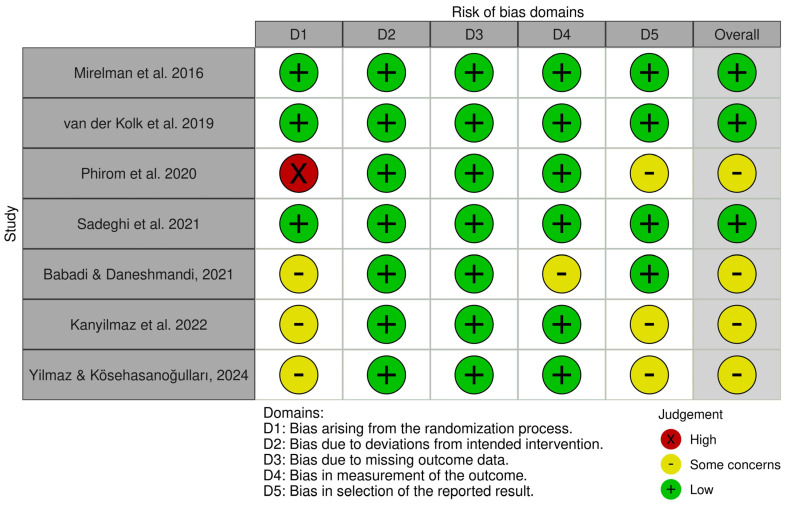
Risk of Bias (RoB-2) assessment of studies [36,37,38,39,40,41,42].

**Figure 3:**
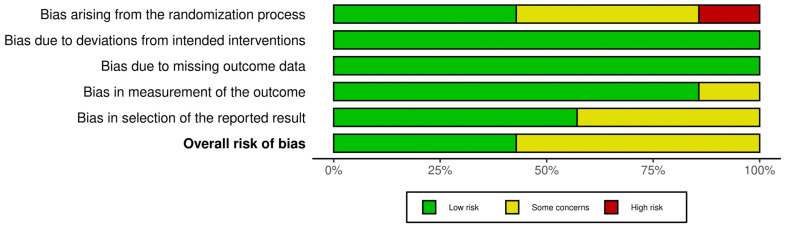
Risk of Bias (RoB-2) assessment of studies.

Overall, the studies exhibited primarily low or moderate risk of bias, which confirms their relatively good methodological quality, although in some cases a cautious interpretation of the results was warranted (Figure 2 and Figure 3).

### 3.4. Balance Outcomes

In several studies, VR-based interventions demonstrated superiority over conventional balance training. In the study by Babadi and Daneshmandi, VR exercises improved balance (measured using, among others, one-leg stance with eyes open/closed, tandem stance, and the Fullerton Advanced Balance Scale) to a similar extent as traditional therapy; both groups showed significant post-intervention improvements, with no statistically significant differences between them [40]. Sadeghi et al. reported that an eight-week combined training program (MIX) incorporating VR elements (interactive games) yielded greater benefits in balance outcomes compared to either traditional balance training or VR alone [38]. In the study by Yılmaz and Kösehasanoğulları, which included female patients with osteoporosis, the use of Nintendo Wii games significantly improved Berg Balance Scale scores (mean BBS ≈ 52.9 at the end of therapy) compared to home-based exercises (*p* = 0.001) [42]. Similarly, in the vestibular rehabilitation study by Kanyılmaz et al., greater improvements in static and dynamic balance (Berg Balance Test) were observed in the VR-supported group compared to the traditional group after a 6-month follow-up period (*p* < 0.05) [41].

### 3.5. Mobility

Sadeghi et al. found that the combined training group (MIX) (VR + exercises) and the VR-only group achieved greater improvements in Timed Up and Go (TUG) performance and gait speed compared to the traditional and control groups [38]. Similarly, in the vestibular rehabilitation study involving VR, significantly greater improvement in TUG performance was reported in the VR group immediately after the intervention (*p* < 0.05) [41]. Among participants with osteoporosis, both groups (Nintendo Wii vs. home-based exercises) significantly reduced their TUG time; however, the final differences between the groups were not substantial [42]. Overall, the results suggest that VR training is at least as effective as conventional training in improving mobility and often provides significant benefits—particularly when VR is integrated with balance exercises or advanced interactive games.

### 3.6. Cognitive Functions

In the study by Phirom et al. (which involved combined physical–cognitive training using interactive games), the VR group showed a significant improvement in MoCA scores compared to the control group (*p* = 0.001), indicating enhanced cognitive function [37]. Simultaneously, the intervention group experienced a reduction in “physiological fall risk,” as measured by the Physiological Profile Assessment (PPA) (*p* = 0.015), and demonstrated better postural stability (*p* = 0.005) [37]. These findings suggest that cognitively engaging VR-based motor training may simultaneously improve both mobility and cognitive performance in older adults.

### 3.7. Risk of Falls

In the study by Mirelman et al. (V-TIME program), the addition of a VR component to treadmill training in older adults significantly reduced the incidence of falls. Six months after the intervention, the VR group exhibited a 42% lower fall rate compared to the non-VR group (incident rate ratio: 0.58; 95% CI: 0.36–0.96; *p* = 0.033) [39]. Phirom et al. demonstrated that participants undergoing VR training showed a statistically significant reduction in PPA scores (indicating lower physiological fall risk) compared to the control group (*p* = 0.015) [37]. In the reviewed studies, VR interventions appear particularly effective in populations at elevated fall risk: individuals with Parkinson’s disease or mild cognitive impairment (MCI; V-TIME), women with osteoporosis, and patients with chronic dizziness. In these groups, VR training improved both clinical measures of balance and mobility, as well as reduced self-reported or objectively measured fall risk (e.g., fear of falling scales in the osteoporosis population).

### 3.8. Analysis of Functional Parameters and Evaluation of Intervention Effectiveness

The study by Mirelman et al. [39] demonstrated that treadmill training with the use of VR (IRR = 0.58; *p* = 0.033) significantly reduced the number of falls in older adults at high risk (including individuals with Parkinson’s disease) compared to treadmill training without VR. The effect persisted over a 6-month follow-up period, particularly within the Parkinson’s disease subgroup (IRR = 0.45; *p* = 0.015). Similarly, Kanyilmaz et al. [41] reported improvements in dizziness-related parameters (emotional DHI subscale) and mobility (TUG) following 3 weeks of VR-supported rehabilitation, with effects maintained at 6-month follow-up. All studies included showed improvements in balance. Babadi and Daneshmandi [40] observed that both VR-based and conventional balance training (CBT) significantly improved SLS, FRT, and TUG test results (*p* < 0.05), with no statistically significant differences between groups. Yilmaz and Kösehasanoğulları [42] demonstrated that VR training using Nintendo Wii led to a 10.2-point improvement on the BBS, compared to a 5.17-point gain in the conventional group (*p* < 0.05). In the study by Sadeghi et al. [38], the combination of balance training (BT) with VR (MIX group) produced the greatest improvement in TUG time (−4.2 s; η^2^ = 0.85; *p* < 0.001), surpassing the effects of standalone VR or BT. The MIX group also achieved a greater increase in muscle strength compared to the VR or BT groups, suggesting a synergistic effect of the combined methods. Additionally, Kochaphan Phirom et al. [37] found that cognitive-motor VR training significantly improved MoCA scores (*p* = 0.001) and reaction time, indicating cognitive benefits. Likewise, van der Kolk et al. [36] reported an increase in VO_2_ max (Δ = 2.4 mL/kg/min; *p* < 0.0001) in patients with Parkinson’s disease, which may contribute to overall functional fitness (Table 3).

### 3.9. Comparison of the Effectiveness of VR and Conventional Methods

The results of the reviewed studies indicate that virtual reality (VR)-based interventions are equally effective or superior to traditional training methods in improving functional outcomes related to fall prevention. In the study by Babadi and Daneshmandi [40], both VR and conventional balance training (CBT) significantly improved performance in the single-leg stance (SLS), Functional Reach Test (FRT), and mobility (TUG), with no statistically significant differences between the groups (*p* > 0.05). Similarly, in the study by Sadeghi et al. [38], the combination of VR and balance training (MIX group) yielded the greatest improvement in TUG performance (−4.2 s; η^2^ = 0.85), surpassing the effects of either VR or CBT alone, suggesting a synergistic interaction between the two approaches. Among patients with Parkinson’s disease, VR-based cycling training produced a greater reduction in motor symptoms (MDS-UPDRS III Δ = −4.2 points; *p* = 0.002) and a significant increase in VO_2_ max (Δ = 2.4 mL/kg/min; *p* < 0.0001) compared to stretching or relaxation exercises, although no significant changes were observed in subjective quality of life [36] (Table 3).

### 3.10. Adherence, Safety, and Long-Term Maintenance of Effects

VR interventions were characterized by high adherence, ranging from 75% to 100% of attended sessions [36,40,42]. Only three studies [36,39,41] included follow-up assessments after the intervention period (6 months). In the study by Mirelman et al. [39], the reduction in fall incidence was sustained, particularly among individuals with Parkinson’s disease (IRR = 0.45; *p* = 0.015). Van der Kolk et al. [36] reported sustained improvements in motor parameters (MDS-UPDRS III) in patients with Parkinson’s disease. Kanyilmaz et al. [41] showed that the benefits in dizziness (DHI) and mobility (TUG) persisted at the 6-month follow-up (*p* < 0.05). Long-term follow-up confirmed the maintenance of effects in fall reduction [39] and alleviation of dizziness symptoms [41], whereas most studies did not include extended follow-up periods (Table 3).

**Table 3:** Detailed analysis of the studies included in the review.

Author	Population	Number of Participants (INT/Control)	Mean Age (±SD)	Intervention	Comparator (Traditional Training)	Duration	Assessed Outcomes	Measurement Tools	Main Findings	Adherence (%)	Follow-Up, Long-Term Effects
Mirelman et al., 2016 [39]	Older adults aged 60–90 years with a high risk of falls (≥2 falls in 6 months): idiopathic (n = 109), mild cognitive impairment (MCI) (n = 43), Parkinson’s disease (n = 130).	Randomization: 302 (INT: 154; Control: 148) ITT Analysis: 282 (INT: 146; Control: 136)	INT: 74.2 (±6.9) Control: 73.3 (±6.4)	Treadmill training + VR (3×/week, 6 weeks): obstacle, pathway, and distractor simulation.	Treadmill training without VR (same intensity and duration).	Intervention: 6 weeks. Follow-up: 6 months.	Primary: Fall incidence rate Secondary: Gait speed, gait variability, endurance (2-min walk), SPPB, SF-36, cognitive function	VR (Microsoft Kinect), Zeno platform, inertial sensors (Opal), NeuroTrax, SPPB, SF-36.	INT vs. Control: IRR = 0.58 (95% CI: 0.36–0.96; *p* = 0.033) Improved gait variability (*p* = 0.018), greater foot clearance (*p* = 0.002).	INT: 92% (16.62/18 sessions) Control: 93% (16.82/18 sessions)	6-month follow-up: sustained improvement in the INT group, particularly among individuals with Parkinson’s disease (IRR = 0.45; *p* = 0.015).
van der Kolk et al., 2019 [36]	Patients with early-stage Parkinson’s disease (Hoehn and Yahr ≤ 2), on stable dopaminergic medication.	Randomization: 130 (INT: 65; Control: 65) ITT Analysis: 125 (INT: 61; Control: 64)	INT: 59.3 (±8.3) Control: 59.4 (±9.3)	Aerobic cycling training with VR (3×/week, 6 months): target heart rate 50–80% HRR, supported by a motivational app and remote supervision.	Stretching and relaxation exercises (3×/week, 6 months): similar support via a motivational app and remote supervision.	Intervention: 6 months. Follow-up: 6 months.	Primary: MDS-UPDRS-III (in the “off” medication state) Secondary: VO_2_ max, quality of life (PDQ-39), cognitive function, number of falls	MDS-UPDRS, VO_2_ max test, motivational app, VR system.	INT vs. Control: difference in MDS-UPDRS-III = −4.2 (95% CI: −6.9 to −1.6; *p* = 0.002) VO_2_ max improvement in INT: Δ = 2.4 mL/kg/min; *p* < 0.0001.	INT: 75% (54/72 sessions) Control: 83% (60/72 sessions)	6-month follow-up: sustained improvement in the INT group (MDS-UPDRS-III), no significant differences in quality of life.
Phirom et al., 2020 [37]	Community-dwelling older adults aged ≥65 years, ambulating independently without assistive devices.	Randomization: 40 (INT: 20; Control: 20) Analysis: 39 (INT: 19; Control: 20)	INT: 70.21 ± 4.18 years Control: 69.40 ± 3.38 years	Cognitive–motor training using Xbox Kinect (3 sessions/week; games requiring stepping, balance, and cognitive tasks).	Educational materials + weekly phone calls to monitor health status (no active training).	12 weeks (36 sessions).	- Fall risk (PPA, TUG) - Cognitive function (MoCA)	PPA (Physiological Profile Assessment), TUG (Timed Up and Go), MoCA (Montreal Cognitive Assessment).	After 12 weeks: INT: Significant improvement in PPA (*p* = 0.002), dual-task TUG (*p* = 0.045), MoCA (*p* = 0.001) vs. control. Control: Worsening of postural sway (*p* = 0.025). INT: Improvement in reaction time, postural sway, executive function and attention.	98.8% (INT: 35.6/36 sessions)	Lack of long-term follow-up; majority of participants were women (82.5%); no data on SRD/MCID for outcomes.
Sadeghi et al., 2021 [38]	Older men aged ≥60 years, community-dwelling, able to walk independently.	Randomization: 64 (BT: 16, VR: 16, MIX: 16, CON: 16) ITT Analysis: 58 (BT: 14, VR: 15, MIX: 14, CON: 15)	- BT: 70.4 (±4.3) - VR: 74.1 (±7.0) - MIX: 70.5 (±5.1) - CON: 72.2 (±7.2)	MIX: Combination of balance training (BT) and VR (3×/week, 8 weeks). VR: Virtual reality games (e.g., Xbox Kinect). BT: Static/dynamic exercises.	CON: Control group without intervention.	8 weeks (3 sessions/week, 40 min/session).	Primary: Muscle strength (quadriceps and hamstrings). Secondary: Balance (one-leg stance, tandem), mobility (TUG, 10mWT)	Biodex isokinetic dynamometer, balance tests (one-leg stance, tandem), TUG, 10mWT.	MIX > VR > BT > CON in improving strength, balance, and mobility (e.g., TUG: MIX −4.2 s; η^2^ = 0.85; *p* < 0.001). VR and BT improved outcomes compared to CON (*p* < 0.05).	- BT: 87.9% - VR: 90.4% - MIX: 92.1%	8 weeks: maintenance of improvements in intervention groups. No long-term follow-up.
Yousefi Babadi & Daneshmandi, 2021 [40]	Older adults (60–75 years) residing in nursing homes (both men and women).	Randomization: 36 (VRT: 12, CBT: 12, Control: 12) ITT Analysis: 36 (no participant loss)	VRT: 66.5 ± 3.8 years CBT: 67.5 ± 3.1 years Control: 66.7 ± 3.2 years	VRT: Training using Xbox Kinect (sports games, e.g., boxing, table tennis). CBT: Conventional balance training (static/dynamic exercises).	Control: No intervention, maintenance of daily activities.	9 weeks (3 sessions/week, 60 min/session).	Primary: Balance (SLS with eyes open/closed, FRT, TUG, FAB)	Tests: SLS, FRT, TUG, FAB.	VRT and CBT: Significant improvement in all balance parameters (*p* < 0.05). No difference between VRT and CBT (*p* > 0.05). Control: No improvement.	100% adherence in VRT and CBT groups (no missed sessions)	9 weeks: maintenance of improvements in intervention groups. No long-term follow-up.
Kanyilmaz et al., 2022 [41]	Older adults (65+ years) with dizziness.	Randomization: 32 (INT: 16, Control: 16) Analysis: 26 (INT: 13, Control: 13)	69.7 ± 6.3 years (all ≥ 65)	VR: Vestibular rehabilitation supported by VR (360° videos using VR goggles + smartphone, e.g., motion simulation in a supermarket).	Conventional vestibular rehabilitation (without VR).	3 weeks (5 sessions/week, 15 sessions).	Dizziness symptoms (VSS) Disability (DHI) Balance (BBT, PST) Mobility (TUG) Anxiety/depression (HAS, GDS) Fear of falling (FES-I)	VSS, DHI, BBT, TUG, FES-I, PST, GDS, HAS. Measurements: before, after 3 weeks, and at 6 months.	After treatment: INT: Significant improvement in emotional DHI and TUG vs. control (*p* < 0.05). At 6 months: INT: Significant improvement in VSS, all DHI subscales, BBT, HAS vs. control (*p* < 0.05). No differences in PST, FES-I, GDS.	87% (2 missed sessions out of 15)	6 months: Maintenance of improvement in VSS, DHI, BBT, HAS in the INT group. No differences in other parameters.
Yilmaz & Kösehasanoğulları, 2024 [42]	Women with osteoporosis (≥45 years).	Randomization: 60 (INT: 30, Control: 30) Analysis: 60 (no participant loss)	WEG: 67 ± 10.64 years HEG: 68 ± 9.06 years	VR: Balance exercises using Nintendo Wii (3 sessions/week, supervised by a physiotherapist).	Conventional home exercises (3 sessions/week).	12 weeks (36 sessions).	Balance (BBS, TUG) Fear of falling (FES)	BBS (Berg Balance Scale), TUG (Timed Up and Go), FES (Falls Efficacy Scale).	After treatment: WEG: Significant improvement in BBS vs. HEG (52.9 ± 3.63 vs. 47.1 ± 2.89; *p* < 0.05). Both groups: Improvement in TUG and FES (*p* < 0.05), but no between-group differences. Difference in BBS (WEG: +10.2 vs. HEG: +5.17; *p* < 0.05).	100% (no missed sessions)	No long-term follow-up. Results reported only after 12 weeks.

SLS—Single-Leg Stance; FRT—Functional Reach Test; TUG—Timed Up and Go Test; FAB—Fullerton Advanced Balance; CBT—Conventional Balance; VRT—Virtual Reality Training.

## 4. Discussion

### 4.1. Balance

Our results indicate that VR-based training is at least as effective as conventional exercises in improving balance among older adults and, in some cases, surpasses traditional methods. In the study by Yousefi Babadi and Daneshmandi comparing 9 weeks of VR balance training to classical balance exercises, significant improvements were found in test results (one-leg stance, FRT, TUG, Fullerton) in both groups, with no significant differences between them—suggesting that VR can provide benefits comparable to conventional training [40]. Conversely, Sadeghi et al. (2021) observed the superiority of VR training over traditional methods in selected aspects—an 8-week intervention showed that the VR group achieved better improvements in balance and functional mobility than the group performing balance exercises alone, although the greatest effects were obtained by combining both methods (mixed training) [38]. Similarly, Yilmaz and Kösehasanoğulları found that the use of Nintendo Wii motion games in women with osteoporosis resulted in significantly greater balance improvement (Berg scale increase averaging 52.9 points) compared to the home exercise group [42]. In vestibular rehabilitation, Kanyılmaz et al. reported that 6 months after a 3-week period of VR-assisted balance training, improvements in static and dynamic balance (Berg Balance Scale) were greater than after traditional rehabilitation [41]. These results align with the literature trend indicating that interactive training can increase engagement of the balance system and provide benefits comparable or superior to classic exercises. For example, in a recent randomized controlled trial, Ghous et al. showed that older adults training with non-immersive VR achieved greater improvements in dynamic balance (measured by DGI) than those undergoing conventional task training [30]. The authors suggest VR as an effective and safe form of balance training for seniors, combining visuospatial stimulation and motor demands in a way that promotes neural adaptation [30].

It is worth noting that not all studies demonstrated VR’s superiority over traditional methods—the effects depended on the intervention’s nature and the studied population. The lack of differences between groups in Yousefi Babadi and Daneshmandi’s study might be due to the relatively small sample size (n = 36) and the use of fairly basic balance exercises in both VR and conventional therapy [40]. Where VR offered additional stimuli (e.g., gaming elements, cognitive tasks, or increased training intensity), clearer benefits were obtained. Sadeghi et al. demonstrated a synergistic effect—the combination of VR and balance training (MIX) yielded the greatest functional gains, exceeding both standalone VR training and conventional exercises [38]. This suggests that VR may be most effective as a supplement to traditional therapy, enriching it with additional stimulation. Overall, the reviewed studies confirm that VR training effectively improves balance in older adults, with the context of VR application (alone or combined with other exercises) and patient characteristics (e.g., Parkinson’s disease) influencing the effect size.

The mechanisms underlying VR-related balance improvements likely involve multisensory stimulation and cognitive engagement during training. Simulated environments require rapid postural responses to changing visual and auditory stimuli, enhancing balance reflexes and sensory integration [43,44]. Repetitive motor tasks in interactive settings have been shown to induce beneficial neuroplastic changes similar to traditional physical activity [31]. Experimental studies confirm increases in neurotrophic factors and changes in neural network activity resulting from “exergaming,” which may translate into better postural control [31,32]. Thus, VR is a promising tool for balance training—particularly in populations requiring additional cognitive stimulation or with attentional deficits affecting stability.

### 4.2. Mobility

Analysis of mobility parameters indicates that VR interventions are equivalent or superior to traditional exercises. Most studies showed that VR training improves gait speed and functional agility at least as effectively as conventional programs, often producing greater reductions in TUG times. In Sadeghi et al., the VR and mixed (VR + balance exercises) groups achieved significantly better TUG results than the conventional training group—the greatest time improvement was in the mixed group (about −4.2 s), significantly larger than VR or balance training alone [38]. Similarly, Kanyılmaz et al. noted faster mobility improvement in the VR group—immediately after 3 weeks of vestibular rehabilitation, TUG performance was significantly better in the VR group than controls (*p* < 0.05), and this effect persisted at 6-month follow-up [41]. In Yilmaz and Kösehasanoğulları’s study, both VR and home exercise groups significantly reduced TUG time, though the final between-group difference was not significant [42]. This suggests that even standard training can improve mobility, but VR is at least equally effective. Importantly, the literature data suggest that VR may outperform traditional training in gait and mobility improvements under certain conditions. Ghous et al. found that seniors training on a non-immersive VR platform achieved better TUG and dynamic gait index (DGI) scores than those in classical peripheral training [30]. The authors regarded VR as a more effective and efficient fall-prevention training form, confirming that virtual elements translate into real improvements in agility and gait safety [30].

Mobility improvement via VR may arise from unique aspects of these interventions. VR applications and motion games often require participants to perform rapid position changes, respond to stimuli, and overcome virtual obstacles, training reaction time, coordination, and divided attention during movement [45,46]. This is similar to dual-task exercises combining motor activity with cognitive tasks—such stimulation fosters better gait automatization and distractor resistance, shortening TUG time and improving gait smoothness [47,48]. In the V-TIME program, adding VR elements to treadmill walking significantly reduced gait variability and improved gait speed in older adults at fall risk compared to treadmill training alone [39]. Although these were secondary outcomes, they indicate practical benefits—integrating cognitive challenges (obstacle avoidance, distractor response) into gait training leads to more stable and faster walking, directly affecting functional mobility and fall avoidance [39].

It should be noted that VR’s superior effects over standard training are not guaranteed in every population. In some groups, baseline fitness may determine a “ceiling” effect—e.g., in highly fit individuals, conventional training already provides maximal benefits, and VR mainly adds entertainment value. In contrast, those with greater deficits (vestibular disorders, Parkinson’s disease) seem to gain added value from VR, learning coping strategies for challenging conditions. The analyzed studies show that populations at higher risk (e.g., those with dizziness) benefit more from VR—consistent with the hypothesis that the more complex the balance/mobility deficit, the more it can be improved through multidimensional VR training. In summary, VR interventions are at least as effective as traditional methods in improving older adults’ mobility and often provide additional benefits in gait speed and quality, especially when requiring simultaneous cognitive engagement.

### 4.3. Cognitive Function

Only a few of the studies included assessed VR training’s effects on cognitive function. Kochaphan Phirom et al. demonstrated that a 12-week interactive cognitive–motor game program (using Kinect sensors) significantly improved cognitive function in older adults compared to no intervention—the mean MoCA score increased significantly in the VR group, while remaining low in controls [37]. Improvements primarily involved executive function and attention, attributed to simultaneous mental and physical stimulation during training [37]. Importantly, this training also reduced physiological fall risk measured by PPA and improved postural balance, indicating that the cognitive component may enhance motor outcomes [37].

Other studies also report beneficial effects of exergaming on seniors’ cognitive abilities, though without clear superiority over conventional activities. For example, Ghous et al. compared 8 weeks of non-immersive VR training with peripheral–stationary training, finding improvements in MoCA scores and quality of life in both groups, with no significant differences [30]. This suggests that well-designed conventional training (including task-oriented and mental stimulation elements) can provide cognitive benefits comparable to VR training [30]. On the other hand, VR exercises are attractive due to entertainment value and require cognitive interaction (movement planning, rapid decision-making), potentially engaging cognitive processes more than routine exercises. According to literature reviews, combining physical activity with cognitive challenges (e.g., multitasking, computer games) improves cognitive function and may delay brain aging [1,49,50]. Moreover, neurophysiological studies indicate that both traditional aerobic exercise and exergaming elevate neurotrophic factors (BDNF) and induce neural plasticity changes responsible for learning [31,32]. Thus, VR training may support cognitive function through biological mechanisms similar to classical physical activity—improving cerebral perfusion, reducing inflammation, and stimulating neuroplasticity [31].

However, evidence on VR’s cognitive effects remains limited. Only one of the studies included directly assessed cognitive changes via standardized testing (MoCA) [37]. Other studies mainly focused on physical parameters related to falls. Future research should include cognitive function measures (memory, executive function, divided attention), especially since cognitive decline is a significant fall risk factor in older adults [23]. Results indicate that VR interventions—especially those designed as integrated cognitive–physical training—can improve both motor and cognitive performance, suggesting a dual benefit: fall risk reduction alongside cognitive support, important for maintaining older adults’ independence. Confirming these findings requires further studies with larger samples and direct comparisons of VR and traditional training regarding neuropsychological changes.

### 4.4. Fall Risk

A key goal of preventive training is to reduce fall frequency in older adults. Although most analyzed studies focused on intermediate parameters (balance, mobility, gait speed, fear of falling), two of the RCTs provided data on actual fall incidence. In Mirelman et al.’s study, involving over 280 high-risk participants, adding a VR component to treadmill walking training resulted in a 42% reduction in fall rate over a 6-month post-training observation compared to treadmill-only training [39]. This difference was statistically significant and especially pronounced in the Parkinson’s subgroup (fall risk decreased by approximately 55%) [39]. These findings demonstrate that training combining motor and cognitive demands (e.g., obstacle avoidance, responding to stimuli while walking) may more effectively prevent falls than physical activity of similar intensity alone. Similar conclusions come from Kochaphan Phirom et al.’s study—although shorter, the intervention significantly reduced physiological fall risk measured by PPA in the interactive VR training group versus controls [37]. This means measurable risk factors (reaction time, muscle strength, postural stability) improved, translating into reduced fall probability.

Other studies assessed subjective and indirect fall risk indicators, also indicating VR benefits. For example, Yilmaz and Kösehasanoğulları observed that both VR and control groups experienced reduced fear of falling (assessed by FES) after 12 weeks of training, with slightly lower final fear levels in the Wii group [42]. Notably, the fact that intensive VR training does not increase fear but rather reduces it is important for senior acceptance of the method. In Kanyılmaz et al.’s study of patients with chronic dizziness, vestibular rehabilitation with VR elements resulted in greater reduction of dizziness severity and improved stability compared to conventional exercises—the effects persisted after 6 months [41].

Our observations align with broader fall prevention research. Classic multicomponent exercise programs (including balance, strength, and gait training) reduce fall risk in older adults by approximately 20–30% [51]. Mirelman et al.’s results suggest VR technology may potentially enhance this effect, possibly by better training of coping abilities in real threat situations (e.g., trips, distractions) [39]. However, caution is warranted in interpretation—as of now, Mirelman’s V-TIME is the largest study clearly showing fall reduction via VR [39]. Other RCTs had sample sizes and observation periods too limited to demonstrate differences in actual falls. Thus, further studies with long-term follow-up (12 months or more) and adequate statistical power are needed to confirm whether VR interventions consistently reduce fall rates in various senior populations. Nevertheless, current data are encouraging—VR appears to be a tool capable of enhancing fall prevention effectiveness, especially for those who do not respond well to conventional exercises alone.

### 4.5. Technology Acceptance, Equipment Requirements, and Therapist Competence

The literature indicates that older adults’ willingness to use VR depends on perceived usefulness, ease of use, and the attractiveness of the training program [52]. Seniors are more likely to engage in VR-based exercises if they perceive tangible benefits and enjoyment from the activity, and if the interface is tailored to their needs. At the same time, potential initial concerns must be taken into account, such as low digital literacy in some older adults and fear of unfamiliar technology. Equally important is the acceptance of VR by therapists—their positive attitude and understanding of VR’s benefits can significantly influence the successful implementation of such programs. If therapists fear that VR equipment will be too time-consuming or complex to operate, or if they are concerned about patient safety, this may hinder adoption of these technologies [53].

Another key issue involves the technical and infrastructural requirements associated with VR therapy. In clinical settings, access to appropriate equipment is essential—from VR headsets or other interfaces to software containing exercises tailored to older users. This entails initial costs for equipment purchase as well as ongoing maintenance (e.g., system updates, technical servicing) [53]. For many rehabilitation centers, implementation costs may represent a significant barrier, especially in the absence of external funding. However, in recent years, more affordable solutions have become available—such as the use of consumer gaming platforms (e.g., Nintendo Wii, Xbox/Kinect)—which opens the possibility of integrating VR even in less-resourced facilities or in home-based rehabilitation programs [41]. Beyond financial constraints, spatial and technical infrastructure requirements also determine the success of implementation. VR therapy often requires a dedicated and safe training area (to minimize the risk of tripping over real-world obstacles during immersion) as well as stable access to electricity and internet connectivity (especially for network-based or telerehabilitation applications). A lack of sufficient space, technical resources, or support infrastructure is frequently cited as a barrier to integrating VR into clinical programs [52].

Another important concern is the safety and tolerability of VR interventions among older adults. While none of the included RCTs reported serious adverse events and dropout rates were low (with adherence rates ranging from 75% to 100%), precautions are nonetheless necessary in clinical practice [41,52]. Older individuals may show reduced tolerance to intensive visual stimuli—some users report temporary dizziness, nausea, or disorientation (commonly referred to as cybersickness). For this reason, initial sessions should be conducted under close therapist supervision, with the option to terminate the exercise if needed. Good practice is to begin with short, low-intensity sessions and gradually increase immersion duration as the patient adapts [54]. Appropriate physical safety measures are also important—the patient should remain within reach of support (e.g., near handrails or wearing stabilizing equipment if needed).

Moreover, the individualization of VR interventions is key to maintaining a balance between effectiveness and safety. VR training programs should be adapted to the functional capacity and health status of the older adult. Different solutions will be appropriate for fit individuals compared to patients with cognitive impairments. For example, in people with significant visual or hearing deficits, systems should incorporate appropriate accommodations (e.g., larger font sizes, additional auditory cues), while in individuals with vestibular disorders, scenarios that may trigger dizziness should be avoided. Adjusting the difficulty level of VR games or training sessions (e.g., slower-moving objects, longer pauses between tasks) helps keep the exercise within the patient’s proximal zone of development while preventing excessive physical or cognitive strain.

### 4.6. Adherence, Safety, and Long-Term Effects

One notable advantage of VR-based interventions appears to be high participant adherence and safety in older adult populations. In the reviewed studies, attendance at VR training sessions was very high—ranging from 75% to nearly 100% of scheduled sessions. By comparison, typical exercise programs for seniors often show lower attendance and engagement (due to monotony or logistical challenges). VR technology seems to increase training attractiveness—participants are more willing to attend sessions, treating them partly as fun or competition. Van der Kolk et al. emphasized that enhancing exercise programs with competitive elements and remote supervision significantly facilitated Parkinson’s patients’ adherence to long-term training regimens [36]. In other words, incorporating interactive games and real-time feedback motivates older adults to exercise regularly, allowing health benefits to accumulate. High adherence was observed not only in lab-supervised interventions but also in home-based VR programs. For example, in van der Kolk et al.’s study, participants performed aerobic cycling at home using a motivational game and were remotely monitored, demonstrating excellent adherence throughout the 6-month program [36].

Regarding safety, VR training was well tolerated by participants, with no serious adverse events related to the technology itself. None of the included studies reported injuries or adverse incidents caused by VR use. In the longer aerobic study by van der Kolk et al., some health events (isolated falls or non-training-related complaints) were reported during the trial, but incidence rates were similar between intervention and control groups [36]. No intolerance symptoms specific to VR (e.g., severe dizziness, nausea, disorientation) were noted, which could theoretically occur in older adults. Ghous et al. described VR intervention as a “safe and feasible” alternative training method for older adults [30].

The issue of long-term maintenance of effects is crucial for evaluating VR intervention utility. Only three reviewed studies included follow-up after program completion (all with 6-month observation). In all cases, benefits gained immediately post-training were maintained after six months. Mirelman et al. showed that the reduced fall rate in the VR group persisted for 6 months post-training, especially in Parkinson’s patients [39]. In van der Kolk et al., Parkinson’s patients undergoing a 6-month aerobic program with gaming elements continued to show motor improvements (UPDRS-III scores) after intervention cessation compared to baseline [36]. Similarly, Kanyılmaz et al. reported that patients maintained improved balance and quality of life (reduced dizziness measured by DHI and better TUG scores) six months after vestibular VR rehabilitation [41]. These observations suggest VR training induces relatively lasting changes, consistent with neuromuscular and behavioral adaptations. It is plausible that engaging, realistic VR scenarios help participants acquire skills used in daily life (e.g., obstacle avoidance, maintaining balance in dynamic environments) and maintain physical fitness for some time post-training.

Of course, lack of monitoring beyond six months leaves questions about the durability of these effects without ongoing training. It is possible that, similar to conventional exercise programs, VR benefits diminish after several months without continued activity. Investigating whether maintenance sessions (e.g., weekly VR sessions or home use of motion games) can prolong effects is important. Nevertheless, current 6-month data demonstrate that VR training produces not only short-term post-intervention improvements but also real, long-term functional changes and fall risk reduction.

### 4.7. Comparison of Results in the Context of Other Meta-Analyses

The findings of our review generally support previous reports but also reveal certain differences. Similar to earlier meta-analyses, we found that VR-based training significantly improves both static and dynamic balance in older adults. This aligns, for example, with the conclusions of Neri et al., who observed better balance outcomes and reduced fear of falling following VR interventions compared to no intervention [25]. Furthermore, our analysis showed that the effects of VR are at least comparable to conventional exercise, which is consistent with the observations by Chen et al. in patients with Parkinson’s disease—where VR was found to match traditional rehabilitation in improving balance and gait [28].

Moreover, our results indicate a potential advantage of VR over conventional training in functional tests (e.g., slightly greater improvements in TUG and BBS in VR groups), as also reported by Ren et al. in seniors with balance impairments [26]. In their study, the difference in TUG time (~0.5 s in favor of VR) was statistically significant. We also observed this effect in our broader review, although it was smaller and borderline significant, which may be due to greater heterogeneity in the included populations [55].

Additionally, we did not find a clear difference between VR and traditional exercises in reducing fall frequency or fear of falling. This result is consistent with the findings of Xu et al., where VR did not outperform standard exercises in alleviating fear of falling [55]. This suggests that psychological factors (such as confidence in mobility) may require longer interventions or additional components, regardless of exercise modality.

Discrepancies between our findings and those of some earlier syntheses may stem from differences in study selection. For instance, the meta-analysis by Saragih et al. focused on VR versus passive control and reported a moderate reduction in fear of falling [33]. In our review—focused on active control groups—the difference in fear of falling between VR and conventional exercise was not significant. This is an important addition to the literature: it indicates that while VR clearly helps reduce fear of falling compared to no intervention, it may not outperform well-designed conventional exercise programs in this regard.

Similarly, Shen et al. observed significant cognitive improvements (in attention and memory) from VR in individuals with mild cognitive impairment (MCI) [34]. Our results also suggest cognitive benefits of VR training; however, not all RCTs we analyzed assessed these parameters, so we interpret this cautiously.

In summary, our findings are generally consistent with existing syntheses regarding the beneficial effects of VR on balance and mobility in older adults [26,28]. At the same time, our review provides new insights: it shows that the effect of VR is comparable to that of traditional exercise in reducing fall risk, and any superiority is mostly observed in selected functional tests. Moreover, we confirm earlier indications of no significant differences in certain areas (e.g., fear of falling, muscle strength) between VR and conventional training [28]. These observations help clarify the role of VR within the spectrum of interventions—as an attractive, effective alternative to traditional exercises, though not necessarily superior in every aspect. Consequently, the review enhances current knowledge by providing a more detailed comparison of VR versus standard physical activity in older populations.

### 4.8. Limitations and Practical Implications

Certain limitations of our analyses and the source studies should be noted. First, the number of available RCTs on this topic remains limited and includes heterogeneous populations (from healthy at-risk older adults to postmenopausal women, etc.). This heterogeneity complicates generalizing conclusions—VR efficacy may vary depending on diagnosis and participant fitness levels. Second, VR intervention protocols differed substantially across studies: ranging from simple balance games to advanced treadmill projection systems and popular consoles (Nintendo Wii, Xbox Kinect). The lack of standardization makes it impossible to identify which VR training type is most effective or whether all are equally good. Particularly problematic is participant and therapist blinding—in interventions as different as VR vs. traditional exercises, full blinding was not feasible, introducing risk of performance bias and expectancy effects. Moreover, small group sizes (often <30 participants per group) limit statistical power and may result in type II errors (failing to detect true differences). For example, in Yousefi Babadi’s study, the lack of difference between VR and exercises might be due to insufficient sample size to detect subtle effects [40]. Another limitation is the short duration of most interventions (3–12 weeks) and the mentioned short or absent follow-up—we do not know whether longer VR programs would yield greater benefits or how long effects last beyond six months.

Considering these limitations, we cautiously interpret that VR-based training may outperform traditional forms of training in certain cases, although this advantage was not confirmed with statistical significance in all studies. Nevertheless, the collected data clearly show that VR is a valuable and effective complement to geriatric rehabilitation, capable of delivering effects comparable to proven methods, often enhancing them with additional benefits (especially in dynamic balance, gait speed, dual-task abilities). Clinically, rehabilitation facilities and therapists should consider integrating VR technology into fall prevention programs for older adults. This applies especially to patients who no longer respond to conventional exercises or quickly become discouraged—VR may increase motivation and engagement, thereby improving overall intervention effectiveness. Moreover, the development of affordable devices (such as motion game consoles and VR goggles) opens the possibility of home training, important for individuals with limited access to rehabilitation centers.

## 5. Conclusions

Interventions using virtual reality proved effective in improving key functional parameters in older adults. The conducted studies indicate significant improvements in postural balance and mobility—seniors after VR training achieved better balance and gait test results, translating into greater stability and independence in daily activities. Additionally, beneficial effects of VR on selected cognitive functions were observed. Fall risk reduction is another key outcome—through improved balance, increased muscle strength, and enhanced confidence, seniors become less prone to dangerous fall incidents. Our research emphasizes the importance of virtual reality as a tool that supports enhancing seniors’ quality of life by helping maintain their functional independence and reducing the burdens associated with falls.

### Future Recommendations

Despite promising results, several research gaps require further exploration. Primarily, longer studies with larger participant groups are needed to assess the durability of obtained effects and confirm that improvements in balance and mobility translate into long-term reductions in fall incidence in real life. It is also important to include more diverse senior populations—such as individuals with varying levels of physical and cognitive abilities or those with neurodegenerative diseases—to determine in which groups VR therapy yields the greatest benefits. Future research should aim to standardize VR intervention protocols (including optimal dose, frequency, and types of virtual exercises), enabling better comparison across studies and development of best practices. Additionally, deeper analysis of VR training’s impact on cognitive functions—such as incorporating dual-task exercises combining physical activity with mental challenges—should be pursued to clarify whether and to what extent virtual reality can support cognitive performance or delay cognitive decline in older adults.

Equally important is translating these findings into clinical practice in geriatric rehabilitation. Guidelines and implementation models for VR therapy should be developed to ensure its effective and safe application in medical facilities and patients’ home environments. This includes addressing logistical and financial issues—providing access to appropriate VR equipment, maintenance, and training therapeutic staff in its use. It is also advisable to investigate acceptance and usability of VR among seniors and therapists: technology must be tailored to older adults’ needs (e.g., simple interfaces, accommodations for vision and hearing limitations, minimizing risks of nausea or disorientation during sessions).

## Figures and Tables

**Table 1:** Inclusion and exclusion criteria based on the PICOS framework.

PICOS	Inclusion Criteria	Exclusion Criteria
Population	mean age of ≥60 years, community-dwelling, ≥1 fall within the past 12 months or a high fall risk score	Participants in severe clinical conditions
Intervention	VR-based training (immersive, non-immersive, exergaming, VR treadmill, telerehabilitation)	Conventional exercises without the use of VR
Comparison	Any exercise program: isolated balance training, strength or aerobic training, multicomponent programs, or no exercise (“usual care”/no exercise)	No control group
Outcomes	At least one of the following: fall incidents; TUG; gait speed; 6MWT; Berg or Y Balance; gait variability; adherence rate (%)	No quantitative measurements of function or falls
Study	Randomized controlled trials; intervention duration ≥ 6 weeks; ≥10 participants	Pilot studies, observational studies, case reports, qualitative studies, reviews

## Data Availability

The original contributions presented in the study are included in the article; further inquiries can be directed to the corresponding author.
